# Cannabidiol Modifies the Formation of NETs in Neutrophils of Psoriatic Patients

**DOI:** 10.3390/ijms21186795

**Published:** 2020-09-16

**Authors:** Piotr Wójcik, Marzena Garley, Adam Wroński, Ewa Jabłońska, Elżbieta Skrzydlewska

**Affiliations:** 1 Department of Analytical Chemistry, Medical University of Bialystok, Mickiewicza 2D, 15-222 Bialystok, Poland; piotr.wojcik@umb.edu.pl; 2 Department of Immunology, Medical University of Bialystok, Waszyngtona 15A, 15-269 Bialystok, Poland; marzena.garley@umb.edu.pl (M.G.); ewa.jablonska@umb.edu.pl (E.J.); 3 Dermatological Specialized Center “DERMAL” NZOZ in Bialystok, 15-453 Bialystok, Poland; adam.wronski@dermal.pl

**Keywords:** psoriasis, cannabidiol treatment, neutrophil traps, NETs, immunity

## Abstract

Psoriasis is associated with increased production of reactive oxygen species which leads to oxidative stress. As antioxidants can provide protection, the aim of this study was to evaluate the effects of cannabidiol (CBD) on neutrophil extracellular trap (NET) formation in psoriatic and healthy neutrophils. Important markers of NETosis were measured in healthy and psoriatic neutrophils after incubation with CBD, lipopolysaccharide (LPS), and LPS + CBD). The percentage of neutrophils undergoing NETosis and the level of NETosis markers (cfDNA, MPO, elastase) were higher in the neutrophils and blood plasma of psoriatic patients, compared to controls. After LPS treatment, all of the markers of NETosis, except elastase, and p47 and citrullinated histones, were increased in samples from healthy subjects and psoriasis patients. CBD reduced the concentrations of NETosis markers. This led to a reduction in NETosis, which was more pronounced in psoriatic neutrophils and neutrophils treated with LPS in both psoriatic and healthy participants. These results suggest that psoriatic patients neutrophils are at a higher risk of NETosis both in vitro and in vivo. CBD reduces NETosis, mainly in psoriatic neutrophils, possibly due to its antioxidant properties. The anti-NET properties of CBD suggest the positive effect of CBD in the treatment of autoimmune diseases.

## 1. Introduction

Psoriasis is the most common autoimmune disease and is usually caused by pathological interactions between lymphocytes and keratinocytes in the skin epithelium [1]. Cytokines, produced by lymphocytes, mediate these interactions. Several cytokines are known to be important in psoriasis pathogenesis. Interferon gamma (IFNγ) increases the proliferation of keratinocytes and activates other immune cells [2,3] and interleukin-22 (IL-22) is responsible for increased keratinocyte proliferation [4]. Tumor necrosis factor alpha (TNFα) [5] and interleukin 17 (IL-17) stimulate the production of chemoattractants in keratinocytes. Chemoattractants (hBD2, S100A9, S100A7, S100A, CCL20) increase migration of leukocytes into the epithelium resulting in inflammation. Inhibitors of TNFα and IL-17 are used as therapeutics for psoriasis, demonstrating the importance of these cytokines in pathogenesis [6,7].

Increased inflammation and the over-proliferation of keratinocytes leads to a characteristic skin change called a psoriatic plaque. Psoriatic plaques can lead to psychological and sociological problems that have a strongly negative effect on the patient’s quality of life. While often associated as a disease of the skin, psoriasis may cause systemic issues [1]. The increased production of cytokines can also over-activate circulating lymphocytes and neutrophils present in the blood [7,8]. It has also been shown that psoriatic granulocytes are characterized by higher production of cytokines and reactive oxygen species (ROS) when compared to healthy individuals [8]. In neutrophils, the over-production of ROS induced by NADPH oxidase may lead to a process called NETosis [9].

It was previously shown that during NETosis antimicrobial proteins such as myeloperoxidase (MPO), neutrophil elastase, and cathepsin G are released into the cytoplasm from azurophilic granules within neutrophils and that NETs also contain them [9]. MPO uses superoxides and hydrogen peroxide generated during an oxidative burst to produce hypochlorous acid and other reactive oxidants. Neutrophil elastase migrates into nucleus, where it degrades proteins that are necessary to maintain chromatin structure, such as H1 histones, which results in chromatin decondensation [10]. At the same time, other histones (H2A, H3, H4) undergo citrullination by peptidylarginine deiminase 4, and the nuclear membrane disrupts. When chromatin is released into the cytoplasm, it is attacked by the antimicrobial proteins produced by neutrophils mentioned above. Finally the cell membrane is disrupted and neutrophil extracellular traps (NETs) are formed [11]. These traps are generated from extracellular DNA associated with antimicrobial proteins originating from neutrophil granules and take the form of a net. The traps bind and destroy pathogens. This is possible because the bacterial cells adhere to DNA and the NETs, in effect, limit their spread. A similar mechanism is observed in other leukocytes, as eosinophils, basophils, mast cells and monocytes are also able to form NETs [12,13,14]. Enhanced NETosis is often observed in other autoimmune diseases besides psoriasis including, systemic lupus erythematosus (SLE) and rheumatic arthritis [12,15]. Since the formation of extracellular traps results in the release of all molecules and proteins contained within cells, including various cytokines, NETosis may be a strong inducer of a pro-inflammatory response in autoimmune diseases. For example, in psoriasis, neutrophils are observed to be the most IL-17 positive skin infiltrating cells, indicating they can be an important source of this and other cytokines [15].

Since neutrophils, like other leukocytes in psoriasis, are constantly over-activated due to pro-inflammatory conditions, they may be at greater risk of various processes including NETosis. These cells may be particularly susceptible to NETosis because psoriasis increases the expression of IL-17, which is a potent activator of NETosis [15]. Therefore, modulation of neutrophil function can be a promising treatment for psoriasis, since current therapies are usually ineffective or induce serious side effects. The psoriasis antibody therapies are very expensive, so they are used only in the most severe cases. Therefore, new compounds are being sought, especially of natural origin, as potential drugs in the treatment of psoriasis.

One potential candidate for therapeutic action seems to be cannabidiol (CBD)—a phytocannabinoid found in *Cannabis Sativa* L., which has no psychoactive effect, but is an anti-inflammatory and antioxidant compound, and its use has shown a positive effect on psoriatic skin cells [16,17]. Since it is known that CBD may reduce neutrophil activity and ROS production therein [18,19], its beneficial effect may be, at least in part, due to the weakening of the skin infiltrating neutrophils. By preventing the production of ROS, CBD may act as a NETosis inhibitor.

Therefore, the aim of this study was to evaluate the differences in NETosis formation between neutrophils obtained from psoriasis patients and healthy control subjects with cannabidiol. Additionally, the effectiveness of cannabidiol was assessed in response to the action of a strong NETosis activator—lipopolysaccharide (LPS).

## 2. Results

**Figure 1:**
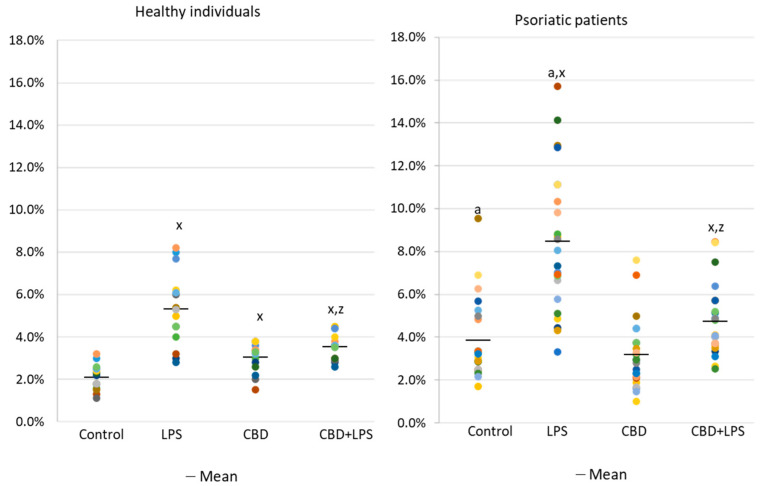
Percentage of neutrophils from healthy subjects (*n* = 14) and psoriatic patients (*n* = 28) undergoing NETosis. after 1h incubation with lipopolysaccharide (LPS), cannabidiol (CBD) and LPS with cannabidiol. a—statistically significant difference between neutrophils from patients with psoriasis vulgaris and healthy subjects *p* < 0.05; x—statistically significant difference between treated neutrophils (with LPS, CBD, or LPS + CBD) and non-treated (control) cells; *p* < 0.05; z—statistically significant difference between neutrophils treated with LPS + CBD and treated only with LPS; *p* < 0.05.

The results show that the percentage of unstimulated neutrophils undergoing NETosis was greater in the psoriasis vulgaris cohort than in healthy control subjects (Figure 1). Incubation with LPS, a known activator of NETosis, increased the number of neutrophils undergoing NETosis in both groups, although the concentration of NETosis was greater in the psoriasis cohort. Treatment with CBD did not cause significant decrease in the number of NETotic cells compared to untreated. In contrast, activation of neutrophils with LPS in conjunction with CBD significantly reduced the percentage of neutrophils undergoing NETosis compared to treatment with LPS alone.

**Figure 2:**
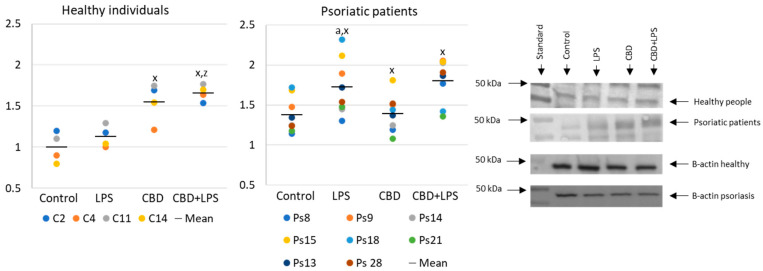
The level of p47 in neutrophils from heathy subjects (*n* = 4) and psoriatic patients (*n* = 8) after 1 h incubation with LPS, cannabidiol and LPS with cannabidiol. a—statistically significant difference between neutrophils from patients with psoriasis vulgaris and healthy subjects *p* < 0.05; x—statistically significant difference between treated neutrophils (with LPS, CBD, or LPS + CBD) and non-treated (control) cells; *p* < 0.05; z—statistically significant difference between neutrophils treated with LPS + CBD and treated only with LPS; *p* < 0.05.

Neutrophils undergoing NETosis show metabolic changes in these cells, assessed by the level of various parameters. The production of the p47 subunit of prooxidative NADPH oxidase, responsible for the generation of superoxide radicals, is strongly increased after LPS administration, especially in neutrophils of psoriatic patients (Figure 2). In this case, CBD appears to enhance p47 production in LPS-treated neutrophils of healthy subjects but does not affect production in psoriasis neutrophils. In neutrophils treated only with CBD decreases in production of p47 was observed in psoriatic patients. In summary, the results suggest that CBD may act as an activator of pro-oxidative NADH of neutrophils in healthy people, but it does not intensify the already existing pro-oxidative conditions in neutrophils from patients with psoriasis.

**Figure 3:**
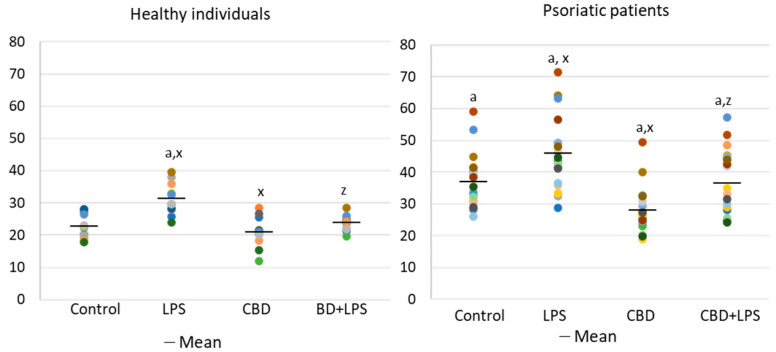
Level of myeloperoxidase (MPO) (ng/mL) in supernatants obtained after 1h neutrophils from healthy subjects (*n* = 14) and psoriatic patients (*n* = 28) incubation with LPS, cannabidiol and LPS with cannabidiol. a—statistically significant difference between neutrophils from psoriasis vulgaris and healthy subjects *p* < 0.05; x—statistically significant difference between treated neutrophils (with LPS, CBD, or LPS + CBD) and non-treated (control) cells; *p* < 0.05; z—statistically significant difference between neutrophils treated with LPS + CBD and treated only with LPS; *p* < 0.05.

In contrast to p47, MPO is released from the cell during NETosis and is therefore assessed in supernatants rather than intracellularly (Figure 3). Since MPO is one of the important markers of NETosis, higher concentration in supernatants of neutrophils obtained from psoriasis patients and in LPS-treated cells suggests an increased NETosis in these groups. On the other hand, in the case of cells treated with CBD, the MPO level is lower, which may inhibit the generation of hypochlorous acid and indicate a decrease in NETosis. Additionally, CBD has an anti-NETotic effect in LPS-treated cells.

**Figure 4:**
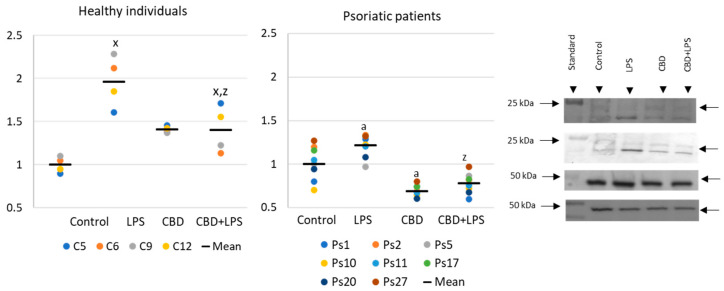
Citrullinated H3-histone expression in human neutrophils from heathy subjects (*n* = 4) and psoriatic patients (*n* = 8) after 1 h incubation with LPS, cannabidiol and LPS with cannabidiol. a—statistically significant difference between neutrophils from patients with psoriasis vulgaris and healthy subjects *p* < 0.05; x—statistically significant difference between treated neutrophils (with LPS, CBD, or LPS + CBD) and non-treated (control) cells; *p* < 0.05; z—statistically significant difference between neutrophils treated with LPS + CBD and treated only with LPS; *p* < 0.05.

Changes in the generation or activity of pro-oxidative proteins in NETosis are also accompanied by histone citrullination (Figure 4). In this case, LPS led to the activation of NETosis, which resulted in increased expression of citrullinated H3-histones. On the other hand, CBD showed anti-NETotic properties, especially in relation to the psoriatic and LPS-activated neutrophils.

**Figure 5:**
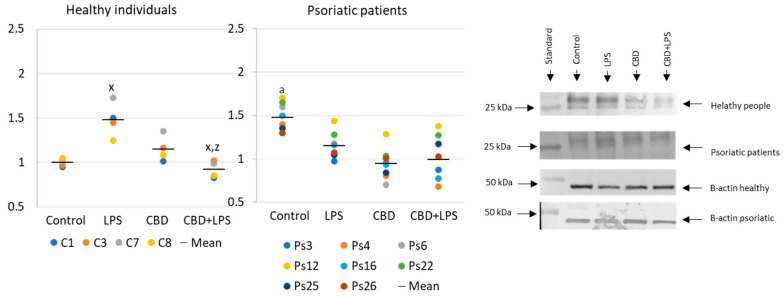
Neutrophil elastase expression in human neutrophils from heathy subjects (*n* = 4) and psoriatic patients (*n* = 8) after 1 h incubation with LPS, cannabidiol and LPS with cannabidiol. a—statistically significant difference between neutrophils from patients with psoriasis vulgaris and healthy subjects *p* < 0.05; x—statistically significant difference between treated neutrophils (with LPS, CBD, or LPS + CBD) and non-treated (control) cells; *p* < 0.05; z—statistically significant difference between neutrophils treated with LPS + CBD and treated only with LPS; *p* < 0.05.

Increased production of neutrophilic elastase is necessary for NETosis and production of elastase was much greater in neutrophils of patients with psoriasis than in healthy subjects, suggesting increased activation of NETosis. CBD appears to have an anti-inflammatory effect as it leads to a reduction in the production of elastase in neutrophils of psoriasis patients. It can therefore be suggested that CBD reduces the effect of LPS on plaque neutrophils (Figure 5).

**Figure 6:**
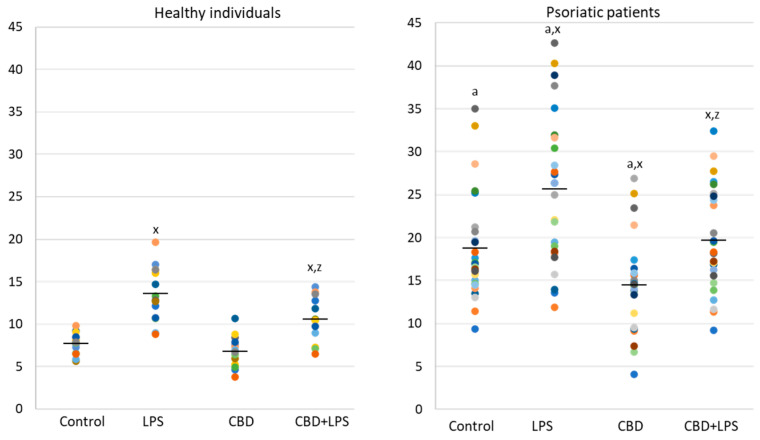
Level of cfDNA (ng/mL) in supernatants obtained after 1h neutrophils, from healthy subjects (*n* = 14) and psoriatic patients (*n* = 28), incubation with LPS, cannabidiol and LPS with cannabidiol. a—statistically significant difference between neutrophils from patients with psoriasis vulgaris and healthy subjects *p* < 0.05; x—statistically significant difference between treated neutrophils (with LPS, CBD, or LPS + CBD) and non-treated (control) cells; *p* < 0.05; z—statistically significant difference between neutrophils treated with LPS + CBD and treated only with LPS; *p* < 0.05.

Since the action of the described enzymes ultimately leads to the release of the DNA, cfDNA it is one of the most reliable markers of NETosis. In the present study, an increase in the concentration of cfDNA was observed in supernatants obtained after incubation of neutrophils from psoriasis patients as well as after incubation of neutrophils with LPS. On the other hand, CBD leads to a reduction in cfDNA levels, suggesting that the effect of CBD on neutrophils ultimately decreases NETosis (Figure 6).

**Figure 7:**
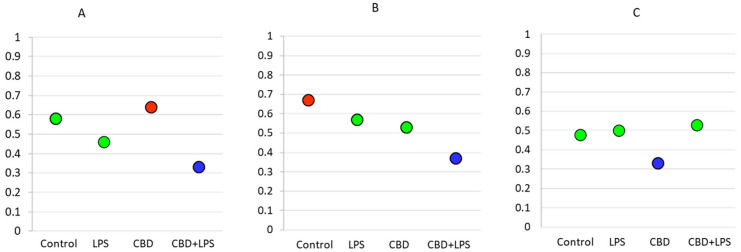
Spearman’s correlation index between PASI and relevant parameters. Blue circle—low correlation (Spearman correlation below 0.4); Green circle—medium correlation (Spearman correlation index from 0.4 to 0.6); Red circle—high correlation (Spearman correlation index from 0.6 to 0.8); (**A**) Percentage of NETotic cells and PASI; (**B**) MPO level and PASI; (**C**) cfDNA and PASI.

As the intensity of NETosis is dependent on the activity of neutrophils, it may be also dependent on the severity of psoriasis. Correlations between the Psoriasis Area Severity Index (PASI)—the most important marker used in clinical practice for the assessment of psoriasis—and in vitro markers of NETosis show that in most cases severity of psoriasis correlates with disease severity (Figure 7). Moreover, it was observed that correlations are still clear also after administration of LPS or CBD in case of percent of neutrophils undergoing NETosis and MPO level. On contrary level of cfDNA correlates with neutrophils stimulated with LPS and LPS + CBD.

**Figure 8:**
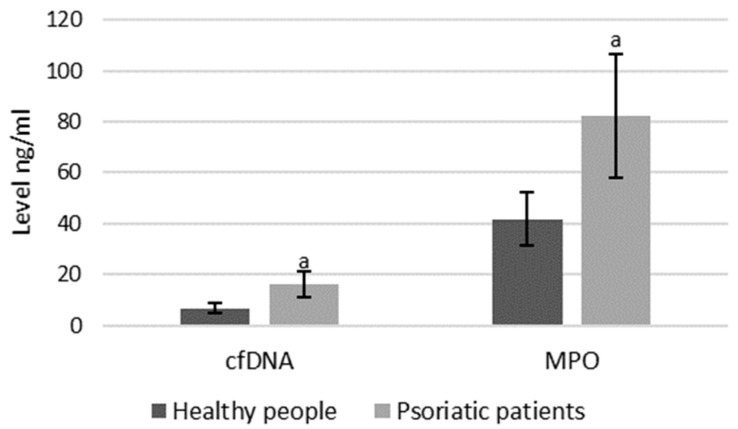
Plasma MPO and cfDNA levels in healthy subjects (*n* = 14) and patients with psoriasis (*n* = 28). a—statistically significant difference between neutrophils from patients with psoriasis vulgaris and healthy subjects; *p* < 0.05.

As mentioned, both MPO and cfDNA are externalized outside the cells, therefore they can also be measured directly in blood plasma, and the level of these parameters may indicate the intensification of NETosis. ELISA assays showed that cfDNA and MPO concentrations are significantly higher in psoriatic patients than healthy subjects, suggesting an increase in NETosis in these patients (Figure 8).

## 3. Discussion

Currently, it is believed that psoriasis is a disease modulated mainly by lymphocytes, especially T lymphocytes. However, neutrophils constitute the largest group of leukocytes and changes in their metabolism and function, such as the higher generation of ROS during activation, increased production of cytokines, and the tendency to undergo NETosis strongly affect systemic functions, which makes them important factors in the pathophysiology of various autoimmune diseases [20,21]. Therefore, granulocytes are gaining more attention as important players in the development of psoriasis. It was previously found that activated granulocytes, under oxidative stress conditions, produce higher amounts of cytokines not only in the skin, but also in the blood of patients with psoriasis [1]. Moreover, it has been shown that neutrophils are involved in the formation of skin lesions during development of psoriasis [8,9].

The literature to date indicates that the increased activity of NADPH oxidase and xanthine oxidase was observed in the entire population of granulocytes in psoriasis patients, which may indicate a much greater oxidative effect in these cells [8]. In neutrophils, activation increases production of NADPH oxidase which generates superoxide radicals, the first reactive oxygen species that are further metabolized to hydrogen peroxide and can be converted into hypochlorous acid by MPO or into a hydroxyl radical in the Fenton reaction, promoting the formation of an oxidative burst [22,23,24]. The concentration of these enzymes correlate with the intensity of NETosis [25]. Our research confirms this previous observation, as elevated levels of NADPH oxidase and MPO are observed in the neutrophils of psoriasis patients, especially when activated by LPS. The increase in the expression of abovementioned pro-oxidative enzymes is accompanied by a higher percent of neutrophils undergoing NETosis observed by us in psoriatic and LPS-activated neutrophils. This is in line with the results seen in previous studies that showed a higher percentage of NETotic cells in psoriasis [26]. This also supports the general observation that in autoimmune diseases enhanced activation of neutrophils leads to enhanced NETosis [27,28]. Moreover, we observe that both the percentage of cells undergoing NETosis and MPO levels correlates with the severity of psoriasis, estimated by the PASI index and these correlations are observed in the case of stimulated and unstimulated cells. This shows that psoriatic cells are more prone to NETosis and this trend increases with the severity of psoriasis. Due to the smaller correlations in LPS-activated cells, this may suggest that neutrophils in the most severe cases of psoriasis are so stimulated that the additional use of activating agents has a slightly smaller effect on their tendency to undergo NETosis.

In normal physiological conditions, an increased level of ROS is usually used to eliminate pathogens, but in autoimmune diseases, the chronic activation of neutrophils leads to a continuous overproduction of ROS. This overproduction contributes to oxidative modification of cellular components including antioxidants (vitamins A, C, E, thioredoxin, glutathione, thioredoxin reductase and glutathione peroxidase) and a reduction in their biological effectiveness. As a result, oxidative stress is an important factor in the pathophysiology of this disease [29]. Oxidative stress also promotes other metabolic changes, including an increase in the production of pro-inflammatory cytokines, which we and other authors previously observed in granulocytes of patients with psoriasis [8,29,30]. In addition, ROS can react with lipids, proteins and nucleic acids to modify their structure and functions, and has been observed with proteins and lipids in psoriatic blood cells [7,8,29,31]. This type of action is believed to lead to the exacerbation of disease symptoms [8].

Extensive oxidative modifications can even lead to apoptosis or necrosis. Moreover, the products of MPO activation are important regulators of the release of neutrophil elastase from granules, as shown by MPO deficiency causes neutrophil elastase to not be released [23]. In our study, higher concentrations of neutrophil elastase were observed in unstimulated neutrophils from psoriasis patients, which confirm higher levels of activation in these cells during disease development. In contrast, neutrophils treated with LPS showed elevated levels of neutrophil elastase in healthy patients, but not in psoriasis patients. As other markers of NETosis show, as well as the percentage of cells undergoing NETosis, psoriatic neutrophils also undergo NETosis after administration of LPS, and the loss of this enzyme is likely due to the fact that it is externalized from the cell.

Neutrophil elastase is essential for NETosis because its action leads to the degradation of nucleus proteins responsible for maintaining chromatin in a condensed form. At the same time, histones are involved in maintaining nucleic acids in nuclei that undergo citrulization. These activities result in the extracellular release of MPO [32] and our study confirms the release of MPO from neutrophils. Higher levels of MPO in plasma and cell supernatants after incubation indicate that psoriasis leads to over-activation of neutrophils and oxidative stress, as reported in our previous work [8], resulting in an intensification of NETosis. On the contrary, in our research, H3 histone citrullination was found to be higher in the healthy people group. This may be due to the fact that histones in psoriasis show a different posttranslational modification profile than in healthy subjects [33], which may be less subject to citrullination. Moreover, in this disease a lower activity of peptidylarginine deiminase 1 is observed [34]. Although this peptidyl-arginine deiminase isoenzyme is not the most important factor in NETosis, its lower activity may partially reduce the expression of citrullinated histones. Therefore, final expression citrullinated H3 histones is lower in psoriatic group. Another confirmation of increased NETosis is the increased level of cfDNA both in supernatants obtained after incubation of neutrophils from psoriatic patients and in the blood plasma. Moreover, it was found that LPS more potently increases the level of cfDNA in relation to the psoriasis patient-derived cells, and cfDNA release correlates with severity of psoriasis, similar like in the case of percentage of neutrophils undergoing NETosis. As NETotic neutrophils are source of cytokines, they are able to affect the Th lymphocyte differentiation profile into domination of Th1 or Th17 [17,35], which are associated with psoriasis [6]. Moreover, as Th17 are source of IL-17, a potent activator of NETosis [36], this can lead to a positive loop in which NETs enhance Th17 domination and Th17 increases NETosis in psoriasis.

As neutrophil and lymphocyte produced cytokines may react with different cells, this may explain why NETs increase the production of antibacterial proteins in psoriasis [26]. Therefore, it may be suggested that NETs may be important factors in the development of psoriasis, especially since LPS, which, like imiquimod, is a TLR4 agonist [37] which causes a strong activation of NETosis [38,39]. Nevertheless, as it is believed that in psoriasis pathological auto-antibodies are not crucial and NETs influence on Th lymphocytes, especially on differentiation, is more important. It is known that mutations in DNAse 1, one of the most important nucleases responsible for degradation of chromatin, lead to impaired NET degradation and is associated with autoimmune diseases [40].

Moreover, NETs were found to exacerbate the course of the disease in the imiquimod-induced mouse model of this disease, since purified NETs activate the cytokine cascade, leading to increased infiltration of immune cells into the skin, leading to an exacerbation of inflammation as measured by levels of pro-inflammatory molecules [41]. As this model is common and frequently used in psoriasis research, these results strongly suggest that NETosis also correlates with the severity of inflammation in psoriatic patients. Moreover, inhibition of peptidylarginine deiminase 4, the key enzyme in the process of histone citrullination, and thus NETosis, leads to an improvement in the condition of the skin in a mouse model [35]. Since this enzyme is key in NETosis, but appears to have very little effect on other neutrophil functions, the improvement in skin condition in this case also indicates that increased NETosis is an important factor that leads to exacerbation of psoriasis symptoms. In addition, during NETosis, DNA stays in contact with pro-oxidative enzymes and the resulting ROS cause oxidative DNA modifications, result in the nucleic acids being resistant to nucleases [42] and making NETs difficult to remove. This allows NETs to affect other cells for a long time. Consequently, therapies based on NETosis inhibition may also be effective in the treatment of psoriasis.

Since NET formation is related to the production and biological function of ROS, and psoriasis is accompanied by oxidative stress, antioxidant compounds may be negative regulators of NETosis in this disease. That is why, for the first time, we examined CBD as a potential anti-NETotic factor in psoriasis. CBD is characterized by a wide spectrum of biological activity, including antioxidant and anti-inflammatory properties, so it is often studied for use in the prevention and treatment of diseases whose development is associated with redox imbalance and inflammation [43]. CBD can regulate redox state directly by affecting elements of the redox system and/or indirectly by interacting with other molecular targets associated with redox system, e.g., G protein-bound receptors [44]. CBD reduces oxidative conditions by preventing the formation of superoxide radicals, generated by xanthine oxidase and NADPH oxidase and by chelating transition metal ions involved in the Fenton reaction to form extremely reactive hydroxyl radicals [45]. CBD also enhances the level of endocannabinoids which activate G protein-bound receptors (cannabinoids and TRPV) [46]. In this study, CBD incubation reduced levels of NADPH oxidase and MPO and consequently reduced NETosis especially in psoriatic neutrophils. However, CBD also decreases MPO concentrations and NETosis in neutrophils obtained from healthy subjects. This indicates inhibition of pro-oxidative enzymes (NADPH oxidase and MPO) by CBD and consequently decreased NETosis.

Moreover, CBD inhibits, at least in part, LPS-dependent alterations in the production of pro-oxidative enzymes, and consequently NETosis, as evidenced by lower levels of cfDNA and the percentage of NETotic cells. This direct action of CBD is supported by the known modulation of cellular antioxidant activity [44]. The antioxidant effect of CBD is also associated with the activation of the redox-sensitive transcription factor Nrf2, which is responsible for the biosynthesis of cytoprotective proteins, including antioxidants, as well as the protection of both non-enzymatic and enzymatic cellular antioxidants [47]. Consequently, CBD prevents oxidative modifications of cellular components, playing an important role in the functioning of NFκB, another redox-sensitive transcription factor. As a consequence, CBD may participate in the regulation of pathological conditions characterized by both redox imbalances and inflammation, such as psoriasis [8,31,48]. Nevertheless, most markers of NETosis (MPO and NETotic cells level) despite adding CBD (in in vitro conditions) still correlate with disease severity. This suggests that although the action of cannabidiol leads to a strong reduction of these markers level in psoriasis, it still unable to completely eliminate the pro-inflammatory neutrophil phenotype in psoriasis, especially in the most severe cases.

Because regulation of cellular redox balance is also dependent on the endocannabinoid system, the action of this phytocannabinoid supports the biological activity of this system. CBD has been shown as activator of endocannabinoid signaling [49], which including the interaction with membrane receptors such as cannabinoid (CB1 and CB2), G protein-coupled receptor 55 (GPR55), ionotropic (TRP) and nuclear (PPAR) receptors that participate in modulation of redox balance and inflammatory conditions [50,51]. Since CB2 receptors, predominating in immune cells, prevent ROS and pro-inflammatory cytokines generation, cannabinoids are thought to be antioxidative and anti-inflammatory agents [52,53].

In fact, psoriasis topical therapy with CBD shows positive effect and leads to alleviation of the skin symptoms of disease [17]. According to current knowledge about CBD action this effect may be mediated by the ability to shift Th differentiation into Th2 lymphocytes [54]. In other diseases models, CBD also shows a positive impact and inhibits the recruitment of neutrophils [55,56], but it is not known if CBD react with neutrophils directly, or whether these changes are due to the anti-inflammatory properties of CBD acting on other cells. The effects of CBD on neutrophils have not been sufficiently studied so far. CBD has recently been observed to inhibit neutrophil migration and their production of ROS, since preincubation of neutrophils with CBD leads to significantly lower ROS levels [18]. As mentioned above, cannabidiol works by activating specific receptors, in the case of immune cells mainly through CB2. Studies with CB2 agonists show that this receptor activation also leads to a reduction in neutrophil migration, but the effect of this receptor on ROS production in neutrophils has not yet been studied [57]. Similarly, activation of the GPR55 receptor present on neutrophils leads to a reduction in the production of ROS, as well as degranulation [58]. On the other hand, in previous studies CBD has not shown an effect on neutrophil viability [18] and its effect on NETosis has not been analyzed. In the study presented here, our results show that CBD decreases rate of NETosis in neutrophils and also production of most of markers of NETosis. Moreover, in the case of psoriasis, CBD may have positive impact not only on NETosis itself, but as CBD is as anti-inflammatory factor, it can inhibit activation of dendritic cells, lymphocytes, and may cause a shift Th lymphocytes into Th2, acting in the opposite manner of NETs, which activate this cells and leads to polarization into Th1 and Th17 [17,35,54,59]. In neutrophils from healthy subjects CBD shows a lower effect than in seen in psoriatic neutrophils. It may be due to the fact, that in psoriatic neutrophils there is increased production of the CB2 receptor, which is responsible for anti-inflammatory effects of CBD [8], potentially explaining why cannabidiol may be more strongly involved in the protection of these cells.

Our results confirmed that psoriatic neutrophils are more susceptible to NETosis than neutrophils from healthy subjects, which may be due to pro-oxidative conditions and the different receptor profiles that are associated with psoriasis. Therefore, the use of CBD has a positive effect and reduces NETosis in psoriatic neutrophils. This mechanism may at least in part account for the positive effect of CBD on psoriasis patients previously observed in vivo.

## 4. Materials and Methods

Twenty-eight patients (15 females and 13 males, mean age 40 years) with a diagnosis of psoriasis vulgaris for at least 6 months and with at least 10% of the total body surface area affected were selected for the study. The severity of psoriasis was assessed with Psoriasis Area Severity Index (PASI) which was calculated according to severity of redness, thickness, and scaliness of the epidermis, weighted by the area of involvement, and mean value of this index was 16.3 [60]. The control group consisted of 14 healthy subjects (8 females and 6 males) with an average age of 42.5 years old. None of the patients or healthy subjects had received topical or oral medications during the 4 weeks prior to the study. Only individuals without any significant comorbidity (liver, kidney, or cardiovascular diseases; cancer; respiratory disorders; and diabetes) and who were not alcohol abusers or smokers were included in the study. The study was approved by the Local Bioethics Committee in the Medical University of Bialystok (Białystok, Poland), No. R-I-002/289/2017. Written informed consent was obtained from all the participants.

Venous blood was collected in tubes containing ethylenediaminetetraacetic acid (K3-EDTA) (Becton Dickinson, Franklin Lakes, NJ, USA). (Plasma was separated by centrifugation (300× *g*, 25 min) collected and stored frozen prior to analysis. To collect neutrophils, blood samples were centrifuged over Polymorphprep™ (300× *g*; 40 min, 4 °C) (Axis-Shield PoC AS, Oslo, Norway) and the granulocyte and leukocyte fractions were collected. Neutrophils were isolated from the granulocyte fraction using a magnetic-activated cell sorting technique and a MidiMACS™ separator equipped with LS columns and CD16 microbeads (Miltenyi Biotec, Bergisch Gladbach, Germany), according to manufacturer’s instructions. Cells were immediately used after collection.

### 4.1. Cells Treatment and Incubation

Collected neutrophils, both from healthy subjects and psoriatic patients, were suspended in Hanks Medium supplemented with fetal bovine serum (FBS) (Biomed, Cracow, Poland) and were incubated at 37 °C with 5% CO_2_ flow for one hour. Incubations were performed in 96 well plates and standardized to contain 100,000 cells in each well for microscopic visualization and 1,000,000 cells in each well for Western blots and ELISA assays. The following treatments were applied:

- Control—no additional compounds
- 10 μg/mL LPS
- 10 μg/mL cannabidiol
- 10 μg/mL LPS + 10 μg/mL cannabidiol

Stock solutions of LPS and cannabidiol were dissolved in PBS and ethanol, respectively.

After incubation, the 96 well plates for Western blots and ELISA assays were centrifuged and the supernatant was removed. The cells and supernatants were stored at −80 °C until used for Western blots and ELISA assay, respectively.

### 4.2. Visualization and Enumeration

Before incubation, FITC conjugated anti-MPO antibodies (Thermo Fisher Scientific, Inc., Waltham, MA, USA) and Hoechst solution were added to stain extracellular MPO and the nucleus, respectively. Stained cells were visualized at 100× magnification using a Nikon Eclipse Ti (Nikon, Tokyo, Japan) fluorescent microscope equipped with standard FITC/TRITC filters, and cells with characteristic changes were enumerated as NETotic. The percentage of NETotic cells compared to total cells was calculated.

### 4.3. Western Blots

Western blot analysis of neutrophil protein expression was performed according to the protocol described in Eissa and Seada [61]. Neutrophils were subjected to sonication and centrifugation (15,000× *g*, 30 min, 4 °C) to obtain a protein fraction which was used for the detection of specific proteins by western blot. Proteins were electrophoretically separated on 10% polyacrylamide gels, transferred to nitrocellulose membranes (0.2 µm pore size), blocked with 5% milk for 1 h (Bio-Rad Laboratories Inc., Hercules, CA, USA) and then washed four times with TBS-T buffer. Washed membranes were incubated overnight with the following primary antibodies: monoclonal against p47, (Santa Cruz Biotechnology, Dallas, TX, USA), β-actin (Sigma-Aldrich; St. Louis, MO, USA), neutrophil elastase (Thermo Fisher Scientific, Inc.; Waltham, MA, USA) and rabbit polyclonal against citrullinated H3 histones (Abcam; Cambridge, MA, USA). After incubation, the membranes were then washed four times with TBS-T and incubated for 2 h with goat polyclonal alkaline phosphatase secondary antibodies (Sigma-Aldrich; St. Louis, MO, USA). Protein bands were visualized using the BCIP/NBT liquid substrate system (Sigma-Aldrich; St. Louis, MO, USA). To compare the proteins concentration between samples, each band intensity was estimated using VersaDoc System and Quantity One software (Bio-Rad Laboratories Inc., Hercules, CA, USA). The results were expressed as a percentage of the expression determined in the control cells.

### 4.4. Examination of MPO Concentration

The MPO concentration in cell supernatants and plasma samples was determined using a commercial ELISA kit (Quantikine ELISA Human Myeloperoxidase Immunoassay, R&D System, Minneapolis, MN, USA), according to the manufacturer’s instructions. This assay employs the quantitative sandwich enzyme immunoassay technique with a microplate pre-coated with monoclonal antibody. The concentration of MPO was determined spectrophotometrically by the intensity of color change in the sample [62].

### 4.5. Examination of cfDNA

The cfDNA concentration in cell supernatants and plasma samples was also determined using a commercial kit (Circulating DNA Quantification Kit, Abcam, Cambridge, MA, USA). This analysis uses a fluorescent method and DNA isolation on spin columns, which uses a solid phase extraction method to quickly purify nucleic acids. Pure DNA is then incubated with assay solution and fluorescence was measured with an excitation wavelength of 480 nm and emission wavelength of 540 nm [63].

### 4.6. Statistics

Statistical analysis was performed using STATISTICA 13.1 (StatSoft Polska, Cracow, Poland). Results were expressed as mean ± SD. Differences between groups were evaluated using Student’s test, and the level of statistical significance was *p* < 0.05. Correlations were evaluated using Spearmen correlation index.

## Abbreviations

CB1	Cannabinoid receptor 1
CB2	Cannabinoid receptor 2
CBD	Cannabidiol
DNA	Deoxyribonucleic acid
IL	Interleukin
LPS	Lipopolysaccharide
MPO	Myeloperoxidase
NE	Neutrophil elastase
NETs	Neutrophil extracellular traps
NADPH	Nicotinamide adenine dinucleotide phosphate
ROS	Reactive Oxygen Species
Th	Helper T lymphocyte
TNF	Tumor necrosis factor
TLR	Toll-like receptors
